# Exploring the use of p53 protein expression as an indicator of oesophageal cancer severity from a high incidence rural area of Africa

**DOI:** 10.4314/ahs.v22i1.39

**Published:** 2022-03

**Authors:** Eugene J Ndebia, Thandazile Ngonyama, Steve Molaoa

**Affiliations:** 1 Department of Human Biology, Faculty of Health Sciences, Walter Sisulu University, Nelson Mandela Drive, 5117, Mthatha, South Africa; 2 Department of Surgery, Faculty of Health Sciences, Walter Sisulu University, Nelson Mandela Drive, 5117, Mthatha, South Africa

**Keywords:** Exploring the use of p53 protein expression, as an indicator of oesophageal cancer severity, from a high incidence rural area of Africa

## Abstract

**Background:**

The expression of p53 has been associated with the severity of other types of cancer. There is scanty information when it comes to oesophageal cancer.

**Objective:**

This study aimed to explore the use of p53 protein expression as an indicator of oesophageal cancer severity from a high-risk incidence in the African rural population.

**Methods:**

Fifty-one patients newly diagnosed with oesophageal cancer were recruited from the endoscopic unit at Nelson Mandela Academic Hospital in Mthatha, South Africa. The serological expression of p53 was measured using the ELISA method and the severity of oesophageal cancer expressed in grade was obtained from the histopathology report from patient's oesophageal biopsies.

**Results:**

We found that the expression of p53 was equally distributed among the histological grades of cancer with the value of 2495 pg/mL ± 1736 pg/ mL for lower grades and 2520 ± 1539 pg/mL for higher grades. Furthermore, we found that the level of p53 expression was equally distributed in patients from grade 1, 2, 3, and 4.

**Conclusion:**

The expression of p53 protein does not vary according to the histological grade of oesophageal cancer in the given population, therefore may not be helpful as a prognostic factor.

## Introduction

The increasing incidence and poor prognosis of oesophageal cancer present a major global public health problem1. More than 80% of deaths from oesophageal cancer occur in developing countries, where the dominant subtype is squamous cell carcinoma. The province of the Eastern Cape in South Africa has been declared as one of the world's regions with the highest rates of oesophageal cancer^2^. Over the past decade, numerous studies have evaluated the prognostic value of p53 protein expression in oesophageal cancer^3^. This protein has become established as a very important marker in tumour development^4^. The expression of p53 has been associated with the severity of other types of cancers including prostate cancer^5^, breast cancer^6^ and colorectal cancer^7^. For oesophageal cancer, there is scanty information on the subject, no consensus has yet been achieved and the available results vary with the region where the study took place. A meta-analysis by Wang et al, 2016^3^ evaluated the expression of p53 and oesophageal cancer prognosis in 36 papers including 4577 patients. They found that p53 expression was significantly associated with a poorer prognosis, bt they did not find a significant association between p53 and histological differentiation. So far, there is a paucity of data on the situation in the Southern Africa population which is mainly rural with a globally wellknown high incidence of oesophageal cancer. Therefore, we conducted this study to explore the possible role of p53 as an indicator of oesophageal cancer severity in a rural African settings.

## Materials and methods

### Settings

The study was carried out at Nelson Mandela Academic Hospital (NMAH) Endoscopic Unit in Mthatha, a rural town in the province of Eastern Cape, South Africa.

### Patients

#### Inclusion criteria

Were included in the study adult patients newly diagnosed with oesophageal cancer by both endoscopy and histopathology. Patients were referred from the endoscopy unit in Nelson Mandela academic hospital.

#### Exclusion criteria

Were excluded from the study patients with other types of cancers, patients with underlined chronic conditions such as diabetes and hypertension.

### Ethical issues

This study was carried out following the code of Ethics of the World Medical Association (Declaration of Helsinki) for experiments involving humans, all participants consented before the study and confidentiality was always observed. Furthermore, the permission to conduct the study was obtained from the Faculty of Health Science Human Research Ethics Committee under reference number 060/2019.

### Study design

This is a cross-sectional study, all newly confirmed oesophageal cancer patients from the endoscopic unit were recruited into the study by convenience as they signed the consent form. Demographic parameters and lifestyle assessment were recorded using a structured questionnaire.

### Laboratory analysis

#### p53 quantification

Venous blood was drawn by a professional nurse, purple top tubes (containing EDTA as an anticoagulant) were used and filled with 8–10 ml of blood per tube. The tubes were then centrifuged at 3200rpm for 10 mins at 4°C. Thereafter, plasma was collected and p53 protein level was evaluated using an Elisa technique which detected both wild and mutated p53 according to the manufacturer guidelines (Elabscience, Inc, USA).

#### Cancer grading

The severity of oesophageal cancer was evaluated from the histology report made at the hospital laboratory by a specialized pathologist from patient oesophageal matched normal and tumour biopsies collected at the time of diagnosis during endoscopy. The severity of the cancer was graded on how abnormal the tumour tissue looked on microscopy as compared to normal tissue. Grading is an indicator of how quickly a tumour is likely to grow and spread. Tumours were graded as 1 to 4 depending on the amount of abnormality. Grade 1 is well differentiated, Grade 2 is moderately differentiated, Grade 3 is poorly differentiated, and Grade 4 is undifferentiated. Grade 1 and Grade 2 tumours tissue appears close to normal. These tumours tend to grow and spread slowly. Grade 3 and Grade 4 tumours do not look normal and tend to grow rapidly and spread faster than tumours of a lower grade.

### Data analysis

Microsoft Excel was used to capture data and Stata15 was used for data management and analysis. Descriptive statistics and frequency distributions were analyzed through means and standard deviations. p53 expression was classified into two groups with a threshold of 2500pg/mL between higher and lower levels. The two groups were formed according to the degree of differentiation: grades 1 and 2 in the first group and grades 3 and 4 in the second group. Univariate and multivariate logistic regression were used to evaluate the correlation between the level of p53 and the grade of oesophageal cancer. A p-value of 0.05 or less was considered statistically significant.

## Results

### Demographic characteristics of the population

A total of 51 patients diagnosed with squamous oesophageal cancer were enrolled in our study. 61 % of the patients were female and 53% were over 65 years of age. Furthermore, 57 % were alcohol drinkers and 53% were smokers and 65% were not educated beyond primary school (Table 1).

**Table 1:** Demographic characteristics of oesophageal cancer patients

Characteristics	Level	Frequency (%)
Gender	Male	20 (39)
	Female	31 (61)
Age	65 and less	24 (47)
	65 and more	27 (53)
Smoking	Yes	24 (47)
	No	27 (53)
Alcohol	Yes	29 (57)
	No	22 (43)
Education	None	6 (12)
	Primary	33 (65)
	High School	12 (24)

### p53 level of expression

The results showed that the mean and standard deviation values of p53 in males were 2728 pg/mL ± 1573 pg/mL, and in females 2369 pg/mL ± 1643 pg/ mL with no statistical difference between the two genders. The level of p53 was found not to be significant between the age groups. In those less than 65years it was 2462 pg/mL ± 1723 pg/ mL and those more than 65 it was 2552 pg/mL ± 1535 pg/ mL. Higher levels of p53 were found in patients with a history of self-induced vomiting at 2774 pg/mL ± 1557 pg/ mL while in those with no history of self-induced vomiting at 1930 pg/mL ± 1619 pg/ mL, with no statistical significance. We also observed that the level of p53 was equally distributed among the histological grades of cancer with the value of 2495 pg/mL ± 1736 pg/ mL for grade 1 & 2 and 2520 ± 1539 pg/mL for grade 3 & 4 (Table 2).

**Table 2:** p53 expression distribution among oesophageal cancer patients

		p53 level (pg/mL)	P-value
Gender	Male	2728 ± 1573	0.7791
	Female	2369 ± 1643	
Age	65 and less	2462 ± 1723	0.4218
	65 and more	2552 ± 1535	
Smoking	Yes	2693 ± 1656	0.776
	No	2346 ± 1581	
Alcohol	Yes	2565 ± 1553	0.6091
	No	2437 ± 1716	
History of self-induced vomiting	Yes	2774 ± 1557	0.9589
	No	1930 ± 1619	
Grade of Cancer	Grade 1&2	2495 ± 1736	0.4784
	Grade 3&4	2520 ± 1539	

Mann – Whitney test (Mean± SD)

### Oesophageal cancer severity

We investigated the relationship between p53 levels and oesophageal cancer severity illustrated by histological grade at from oesophageal biopsies at the time of diagnosis. The results showed there is no evidence of a relationship between p53 and the concerned variables (Table 3). We further proceeded with the analysis by using logistic regression analysis. This demonstrated that the probability of having higher levels of p53 is 47% more in patients who have high grade cancer compared to those with low grade cancer (Table 4). Nevertheless, none of these differences achieved statistical significance.

**Table 3:** Bivariate analysis of the association between the level of p53 and oesophageal cancer severity

		Low p53 level	High p53 level	P-value
Grade of Cancer	Grade 1&2	12	10	0.492
	Grade 3&4	13	16	

Chi Square test

**Table 4:** Multivariate analysis of the association between the level of p53 and oesophageal cancer severity

		OR (adjusted)	95% CI	P-value
Grade of cancer	Grade 1&2	1	0.43–5.01	0.534
	Grade 3&4	1.47		

OR: Odd ratio; CI: Confidence interval

## Discussion

Oesophageal carcinogenesis is a complex process involving or dependent on several parameters, including genetic or environmental factors. There is no consensus on the precise pathogenesis of oesophageal cancer. Numerous studies indicate that it is multifactorial8. We evaluated the level of expression of both mutant and wild type of p53 in fifty-one oesophageal cancer patients and the results revealed that the amount of p53 expressed does not vary according to gender, age, smoking, alcohol intake and most importantly it does not vary according to the severity of oesophageal cancer at diagnosis. A trend to high levels of p53 was found in patients with a history of self-induced vomiting but this was not statistically significant.

According to Melling et al. 2019^9^, only a few studies have assessed whether the accumulation of p53 affects oesophageal cancer progression. In our study, the level of p53 was not associated with oesophageal cancer grade. These results are supported by other studies published by Kato et al., 2001^10^, Chino et al., 2001^11^ and Akshatha et al., 2016^12^ demonstrating that the level of p53 is not related to oesophageal cancer severity. Conversely, other published studies by Huang et al., 2014^13^, Hanel et al., 2012^14^ and Melling et al., 2019^9^ have found a significant association with the level of p53 and the severity of oesophageal cancer with higher levelf p53 found in advanced grade. These conflicting results on the relationship between the level of p53 and the grade of oesophageal cancer observed in the literature open the way for further investigations on the matter.

Our study showed a constant level of p53 throughout the grades of oesophageal cancer in patients after its initial surge, suggesting that the level of p53 increases only at early carcinogenesis of the oesophagus as seen in the literature and its level does not change throughout the development of the disease. This constant observed level of p53 is crucial in confirming the reason why in this study there is no statistically proven relationship between the variation of p53 and the severity of oesophageal cancer represented by the histological grades of the disease. While the molecular mechanism of this initial surge is still unclear, this may help in detecting early case of oesophageal cancer in the studied population.

## Conclusion

In summary, our findings showed that the level of expression of p53 does not vary with the severity of oesophageal cancer after its initial surge in the study population. As expected we found an increased level of p53 protein in our patients confirming that p53 gene may be useful as an oesophageal cancer diagnostic tool, but cannot be used as a marker of oesophageal malignancy severity.
